# Incidence of adverse mental health outcomes after sleeve gastrectomy compared with gastric bypass and restrictive bariatric procedures: a retrospective cohort study

**DOI:** 10.1002/oby.23757

**Published:** 2023-06-27

**Authors:** Priya Sumithran, Leo Roberts, Ian D. Caterson, Robyn M. Brown, Matthew J. Spittal, Wendy A. Brown

**Affiliations:** ^1^ Department of Medicine (St Vincent's) University of Melbourne Fitzroy Victoria Australia; ^2^ Department of Endocrinology Austin Health Heidelberg Victoria Australia; ^3^ Department of Surgery Monash University Melbourne Victoria Australia; ^4^ Centre for Mental Health, Melbourne School of Population and Global Health University of Melbourne Parkville Victoria Australia; ^5^ Boden Initiative, Charles Perkins Centre, University of Sydney New South Wales Australia; ^6^ Department of Biochemistry and Pharmacology University of Melbourne Parkville Victoria Australia; ^7^ Department of Surgery Alfred Hospital Melbourne Victoria Australia

## Abstract

**Objective:**

This study examined rates of suicide and hospitalization with psychiatric diagnoses after sleeve gastrectomy compared with gastric bypass and restrictive procedures (gastric banding/gastroplasty).

**Methods:**

This was a longitudinal retrospective cohort study comprising all patients who underwent primary bariatric surgery in New South Wales or Queensland, Australia, between July 2001 and December 2020. Hospital admission records, death registration, and cause of death records (if applicable) within these dates were extracted and linked. Primary outcome was death by suicide. Secondary outcomes were admissions with self‐harm; substance‐use disorder, schizophrenia, mood, anxiety, behavioral, and personality disorders; any of these; and psychiatric inpatient admission.

**Results:**

A total of 121,203 patients were included, with median follow‐up of 4.5 years per patient. There were 77 suicides, with no evidence of difference in rates by surgery type (rates [95% CI] per 100,000 person years: 9.6 [5.0–18.4] restrictive, 10.8 [8.4–13.9] sleeve gastrectomy, 20.4 [9.7–42.8] gastric bypass; *p* = 0.18). Rates of admission with self‐harm declined after restrictive and sleeve procedures. Admission with anxiety disorders, any psychiatric diagnosis, and as a psychiatric inpatient increased after sleeve gastrectomy and gastric bypass, but not restrictive procedures. Admissions with substance‐use disorder increased after all surgery types.

**Conclusions:**

Variable associations between bariatric surgeries and hospitalization with psychiatric diagnoses might indicate distinct vulnerabilities among patient cohorts or that differing anatomical and/or functional changes may contribute to effects on mental health.

**Study Importance**

**What is already known?**

- Bariatric surgery is associated with adverse mental health outcomes for some patients.
- Rates of adverse mental health outcomes are higher after Roux‐en‐Y gastric bypass than after adjustable gastric banding and vertical banded gastroplasty.
- Sleeve gastrectomy is now the most commonly performed bariatric procedure worldwide, but little is known about its association with mental health outcomes.

**What does this study add?**

- We found no evidence that rates of suicide after surgery differed among procedures (rates [95% CI] per 100,000 person years: 9.6 [5.0–18.4] restrictive, 10.8 [8.4–13.9] sleeve gastrectomy, 20.4 [9.7–42.8] gastric bypass, *p* = 0.18).
- Presurgery to postsurgery changes in rates of hospital admission with several psychiatric diagnoses differed by surgery type. Rates of admission with self‐harm declined after restrictive and sleeve procedures; admission with anxiety disorders, any psychiatric diagnosis, and as a psychiatric inpatient increased after sleeve gastrectomy and gastric bypass; and admissions with substance‐use disorder increased after all surgery types.

**How might these results change the direction of research or the focus of clinical practice?**

- Bariatric surgery candidates should be informed preoperatively of the risk of adverse psychiatric outcomes, which varies according to procedure type.
- Future research is needed to understand whether differential effects on mental health may be related to distinct vulnerabilities among different cohorts of patients undergoing each procedure and/or to procedure‐specific changes in gut‐brain signaling.

## INTRODUCTION

Bariatric surgery is an effective treatment for obesity, resulting in sustained total weight loss of 25% to 30% and improvement or resolution of complications of excess weight [1]. Although most studies have reported improvements in overall quality of life and symptoms of depression after bariatric surgery [2], there are also concerns about adverse mental health outcomes for some patients. Rates of suicide after bariatric surgery are up to four times higher compared with the general population [3], and higher rates of self‐harm, mental health‐service use, and alcohol‐use disorder have also been reported postoperatively [4, 5, 6].

The reasons for this are not known, but there is a high preoperative prevalence of trauma, psychiatric conditions, and medication use in people undergoing bariatric surgery compared with the general population [7]. It is notable that rates of adverse mental health outcomes are higher after gastric bypass than after “restrictive” bariatric procedures (adjustable gastric banding and vertical banded gastroplasty) [4]. This could indicate differences in the underlying psychological vulnerability of these patient cohorts or that factors specific to each procedure might differentially affect mental health. For example, gastric bypass and gastric banding have several differences in their effects on the production and release of gut hormones, gut microbial composition, bile acid circulation, and vagal signaling, all of which have been shown to influence behavioral correlates of mood states in animal models [8].

To date, studies of psychiatric outcomes after bariatric surgery have largely analyzed surgeries as a single group or focused on gastric bypass in comparison with restrictive procedures [3, 5], reflecting the previous dominance of these procedure types. Little is known about whether sleeve gastrectomy is also associated with adverse mental health outcomes. This is an important knowledge gap because sleeve gastrectomy is now the most commonly performed primary bariatric procedure globally. Additionally, characterizing the pattern of these adverse effects after different procedures may inform hypotheses about their likely underlying mechanisms. Therefore, this study aimed to examine rates of suicide and hospitalization with psychiatric diagnoses after sleeve gastrectomy compared with gastric bypass and restrictive bariatric procedures. Based on similarities among sleeve gastrectomy and gastric bypass in their indications, weight outcomes, and effects on circulating gut hormones, we hypothesized that the incidence of suicide would be higher after sleeve gastrectomy and gastric bypass compared with restrictive procedures and that the risk of hospitalization for psychiatric outcomes would increase after sleeve gastrectomy and gastric bypass (compared with preoperative risk).

## METHODS

### Study design and setting

We undertook a longitudinal, retrospective, population‐based cohort study of all patients who underwent bariatric surgery in any public or private hospital in New South Wales (NSW) or Queensland, Australia, between July 1, 2001, and December 31, 2020. Patients were followed for up to 19.5 years to observe suicide, self‐harm, and mental health outcomes. Around half of all bariatric surgeries performed in Australia were conducted in these two states [9].

### Data sources

Hospital admission data were obtained from Admitted Patient Data Collections in each state, and death registration and cause of death information (where applicable) were obtained from the Registries of Births, Deaths and Marriages in each state. The Admitted Patient Data Collections provide complete coverage of treatment in all public and private hospitals in their respective states. Similarly, the death registries provide complete coverage of deaths occurring in each state, noting that any deaths occurring outside these states (e.g., in another state or country) are excluded. Data linkage was conducted by the Centre for Health Record Linkage in NSW and the Statistical Services Branch of Queensland Health. Approval was obtained from the NSW Population and Health Services Research Ethics Committee (2020/ETH00760) before the deidentified data were released to investigators. Consent from individual participants was not required.

### Inclusion and exclusion criteria

The inclusion criteria were any patient admitted to a public or private hospital between July 1, 2001, and December 31, 2020, in NSW or Queensland for a primary bariatric procedure. We excluded patients aged <18 years or whose earliest procedure was a reversal or revision (indicating that they had already had bariatric surgery prior to the study period or outside NSW/Queensland) or a temporary endoscopic procedure. Because of small numbers (*n* = 388), patients who underwent bariatric procedures coded as “other malabsorptive” were also excluded.

### Data set construction, variables, and outcomes

For each participant, all other hospital admission records (i.e., those not related to bariatric surgery) and death registration and cause of death records (if applicable) within the same dates were extracted from the admitted patient data collections and death registries. Hospital admission data were linked to death registration and cause of death data using an individual linkage key created by the agency in each state. We defined the index bariatric surgery as the earliest bariatric procedure in the study period, determined using the Australian Classification of Health Intervention (ACHI) codes (Supporting Information Table S1). Surgical procedures were categorized as either restrictive, sleeve gastrectomy, gastric bypass, other malabsorptive, or temporary endoscopic procedures. Because unique ACHI codes for gastroplasty, gastric banding, and sleeve gastrectomy were introduced only in July 2013, we categorized ACHI codes for “gastric reduction” as a restrictive procedure (adjustable banding or gastroplasty) prior to July 1, 2007, and as sleeve gastrectomy between July 1, 2007, and July 1, 2013, based on trends of bariatric surgery in Australia over that period [10].

The primary outcome was death due to suicide. This was extracted from cause of death records using the underlying cause of death field. Suicide was defined as a death associated with one of the following *International Classification of Diseases, 10th Revision* (ICD‐10) codes: intentional self‐harm (X60‐X84), event of undetermined intent (Y10‐Y34), or sequelae of intentional self‐harm (Y87.0).

Secondary outcomes were extracted from hospital records using primary and contributory diagnosis fields, coded using ICD‐10 codes. ICD coding in Australia is done by trained coders and based on information contained in the medical record. Our secondary outcomes were admissions with self‐harm (X60‐X84, Y10‐Y34, X87.0); mental and behavioral disorders due to psychoactive substance use (F10‐F19); schizophrenia, schizotypal, and delusional disorders (F20‐F29); mood disorders (F30‐F39); anxiety, dissociative, stress‐related, somatoform, and other nonpsychotic mental disorders (F40‐F48); behavioral syndromes associated with physiological disturbances and physical factors (F50‐F59); and disorders of adult personality and behavior (F60‐F69). We also coded a variable for hospital admission with any of these diagnoses and for admission as a psychiatric inpatient.

We used this information to construct a consolidated person‐level data set, which included date of first admission to hospital and last follow‐up (December 31, 2020, or date of death), date and type of bariatric surgery, sex and age (in 5‐year categories) at index bariatric surgery, state (NSW or Queensland), and hospitalization with psychiatric diagnoses between July 1, 2001, and the date of index bariatric surgery. The data included a binary variable representing death due to suicide and included counts of admissions for each of the secondary outcomes before and after index bariatric surgery. For our analysis of secondary outcomes, we reshaped this data set into a long form with two rows of data per person: one row pertaining to presurgery values on the secondary outcomes and the exposure time and the other row to the corresponding postsurgery values.

### Statistical analyses

We report participant characteristics at the date of index bariatric surgery using counts and percentages. We estimated the crude suicide rate (per 100,000 person years) by surgery type and by sex, age group, state, and history of admission with psychiatric illness. We performed a fully parametric survival analysis to estimate the risk of suicide by surgery type, including covariates of sex, age group, state, and previous admission with psychiatric illness (all at date of bariatric surgery). We used the Weibull distribution to model the underlying distribution of time. Person time was calculated from the date of surgery until December 31, 2020, or date of death (whichever came first).

For secondary outcomes, we report crude rates for each outcome before and after surgery using the person time from the whole sample in the calculation of the rates. We then performed a fixed‐effects negative binomial regression to estimate the within‐person change in each outcome by surgery type. Fixed‐effects models measure within‐person changes in the outcome and control for both measured and unmeasured individual time‐invariant factors [11], with each person acting as his or her own control. To estimate change in outcomes following surgery, the models included an interaction between surgery type and period (presurgery and postsurgery). We also included person time in each period as an offset term. We report the rate ratios (RRs) for the within‐person change in each outcome presurgery to postsurgery by surgery type and the *p* value for this interaction. We also report the rate difference from the models using marginal effects. Because fixed‐effects models require both the outcome variable and the exposure variables to vary over time (presurgery vs. postsurgery), any observations in which the outcome variable was time‐invariant were excluded from these analyses. This predominantly affected the data from patients who had no admissions with the outcome before and after surgery.

We performed two additional analyses to test the sensitivity of our findings. First, because ACHI coding for bariatric surgery changed during the study period, we repeated the analyses of secondary outcomes including only patients whose surgery was performed after July 1, 2013 (when a unique code for sleeve gastrectomy was introduced). Second, because some participants had more than one bariatric procedure (revisional surgery) during the study period, we repeated the analysis of secondary outcomes excluding those who had revisional surgery.

Our primary analysis was undertaken on a complete case basis owing to the very small amount of missing data (*n* = 22 for age and *n* = 3 for sex). Secondary analyses included only those cases in which the outcome differed between the pre‐ and postsurgery periods, as described earlier. All data management was undertaken in R (version 4.1.2), and all statistical analyses were performed in Stata (version 16.1, StataCorp LLC). We interpret *p* values as a measure of the strength of the evidence against the null hypothesis, with values between 0.01 and 0.05 indicating weak evidence, values between 0.001 and 0.01 indicating moderate evidence, and values ≤ 0.001 indicating strong evidence.

## RESULTS

During the study period, a total of 121,203 patients had a primary bariatric procedure in NSW or Queensland (Table 1). Most were female (76%) and aged between 20 and 59 years (90%). Sleeve gastrectomy was the most common procedure (85%). Median follow‐up time after surgery was 4.5 years per patient (range 0.03–19.5 years; median, restrictive 14.1 years, sleeve gastrectomy 4.4 years, gastric bypass 2.3 years). A total of 85,067 procedures were performed after July 1, 2013, when a new code for sleeve gastrectomy was introduced. During the study period, a total of 15,140 procedures were a second (revisional) bariatric surgery.

**TABLE 1:** Participant characteristics

	All patients	Restrictive surgery	Sleeve gastrectomy	Gastric bypass	*p* value
Total, *n* (%)	121,203 (100%)	7866 (100%)	103,484 (100%)	9853 (100%)	
Sex, *n* (%)					<0.001
Female	92,771 (76.5%)	6189 (78.7%)	79,157 (76.5%)	7425 (75.4%)	
Male	28,429 (23.5%)	1677 (21.3%)	24,324 (23.5%)	2428 (24.6%)	
Age group (y), *n* (%)					<0.001
18–39	49,530 (40.9%)	3038 (38.6%)	43,428 (42.0%)	3064 (31.1%)	
40–59	61,395 (50.7%)	4107 (52.2%)	51,729 (50.0%)	5559 (56.4%)	
≥60	10,256 (8.5%)	720 (9.2%)	8306 (8.0%)	1230 (12.5%)	
State, *n* (%)					< 0.001
New South Wales	64,037 (52.8%)	6482 (82.4%)	53,891 (52.1%)	3664 (37.2%)	
Queensland	57,166 (47.2%)	1384 (17.6%)	49,593 (47.9%)	6189 (62.8%)	
Previous hospitalization with psychiatric diagnosis, *n* (%)					<0.001
No	120,644 (99.5%)	7826 (99.5%)	103,059 (99.6%)	9759 (99.0%)	
Yes	559 (0.5)	40 (0.5%)	425 (0.4%)	94 (1.0%)	
Follow‐up time (y), median (min, max)	4.5 (0.03, 19.5)	14.1 (0.03, 19.5)	4.4 (0.05, 13.5)	2.3 (0.05, 19.5)	

*Note*: Some cells do not sum to the totals because of missing data (*n* < 5 [<0.01%] for sex; *n* = 22 [0.02%] for age group). *P* values for comparisons across surgery types.

Abbreviations: max, maximum; min, minimum.

There were 77 suicides during the follow‐up period (Table 2). Nine were among patients who had a restrictive procedure (9.6 suicides per 100,000 person years), sixty‐one were in patients who had a sleeve gastrectomy (10.8 per 100,000 person years), and seven occurred in patients who underwent gastric bypass surgery (20.4 per 100,000 person years). In survival analysis, after adjustment for covariates, there was no evidence that suicide rates differed by surgery type (*p* = 0.18).

**TABLE 2:** Risk of suicide by surgery type and other demographic and clinical factors

	Number of suicides	Crude suicide rate per 100,000 (95% CI)	Adjusted hazard ratio (95% CI)	*p* value
Surgery type				0.18
Restrictive	9	9.6 (5.0 to 18.4)	Reference	
Sleeve gastrectomy	61	10.8 (8.4 to 13.9)	1.35 (0.65 to 2.78)	
Gastric bypass	7	20.4 (9.7 to 42.8)	2.55 (0.93 to 7.01)	
Sex				<0.001
Female	37	7.6 (5.6 to 10.3)	Reference	
Male	40	22.6 (16.4 to 31.3)	2.96 (1.88 to 4.64)	
Age group (y)				0.70
18–39	31	11.3 (8.0 to 16.1)	Reference	
40–59	37	10.3 (7.5 to 14.3)	0.84 (0.52 to 1.36)	
≥60	9	15.1 (7.8 to 29.0)	1.06 (0.50 to 2.25)	
State				0.20
New South Wales	52	12.5 (9.5 to 16.4)	Reference	
Queensland	25	9.1 (6.1 to 13.4)	0.73 (0.45 to 1.19)	

*Note*: *p* values for differences in incidence of suicide by surgery type and other baseline factors. Adjusted hazard ratio refers to adjustment for all other variables in the table and previous hospitalization with a psychiatric diagnosis.

Crude rates for each of the secondary outcomes are shown in Supporting Information Table S2. Within‐person changes in rates of secondary outcomes after bariatric surgery differed by type of surgery for six of the nine outcomes (Table 3). Restrictive procedures and sleeve gastrectomy (but not gastric bypass) were associated with reductions in hospitalization with self‐harm (RR = 0.64 restrictive, RR = 0.88 sleeve, and RR = 1.12 gastric bypass; *p* = 0.02 for interaction). Restrictive procedures were associated with a reduction in hospitalization with mood disorders (RR = 0.63), any psychiatric diagnosis (RR = 0.78), and admission as a psychiatric inpatient (RR = 0.77) after surgery. There was an increase in hospitalization with substance use following all surgery types (RR = 1.52 restrictive, RR = 2.26 sleeve, and RR = 3.23 gastric bypass, *p* < 0.0001). Only gastric bypass was associated with an increase in hospital admissions with mood disorders (RR = 1.24), whereas both sleeve gastrectomy and gastric bypass were associated with increases in admissions with anxiety disorders (RR = 1.17 sleeve, RR = 1.70 bypass), any psychiatric diagnosis (RR = 1.16 sleeve, RR = 1.66 bypass), and admission as a psychiatric inpatient (RR = 1.23 sleeve, RR = 1.50 bypass).

**TABLE 3:** Presurgery and postsurgery hospitalization for secondary outcomes, fixed‐effects rate ratios, and rate differences

Outcome by type of bariatric surgery	Fixed‐effects rate ratio (95% CI)	Fixed‐effects rate difference per 1000 person years (95% CI)	*p* value for interaction
*Self‐harm, n = 3482*			0.02
Restrictive	0.64 (0.47 to 0.88)	−40.9 (−80.8 to −1.1)	
Sleeve	0.88 (0.81 to 0.95)	−11.3 (−18.4 to −4.2)	
Bypass	1.12 (0.88 to 1.43)	8.5 (−10.3 to 27.4)	
*Substance‐use disorder, n = 5928*			<0.0001
Restrictive	1.52 (1.18 to 1.95)	17.5 (4.4 to 30.5)	
Sleeve	2.26 (2.13 to 2.40)	41.5 (35.9 to 47.1)	
Bypass	3.23 (2.75 to 3.78)	90.1 (60.5 to 119.7)	
*Schizophrenia disorders, n = 803*			0.07
Restrictive	0.63 (0.42 to 0.95)	−42.7 (−86.9 to 1.5)	
Sleeve	1.06 (0.91 to 1.23)	3.6 (−6.1 to 13.4)	
Bypass	0.97 (0.61 to 1.56)	−2.3 (−39.3 to 34.6)	
*Mood disorders, n = 7994*			<0.0001
Restrictive	0.63 (0.53 to 0.75)	−18.6 (−26.3 to −10.8)	
Sleeve	1.02 (0.97 to 1.07)	0.9 (−1.3 to 3.1)	
Bypass	1.24 (1.07 to 1.44)	12.1 (3.4 to 20.8)	
*Anxiety disorders, n = 8934*			<0.0001
Restrictive	0.95 (0.79 to 1.14)	−1.7 (−7.7 to 4.2)	
Sleeve	1.17 (1.11 to 1.23)	7.2 (4.8 to 9.5)	
Bypass	1.70 (1.48 to 1.96)	33.2 (21.4 to 45.0)	
*Behavioral disorders, n = 733*			0.11
Restrictive	0.42 (0.24 to 0.75)	−13.0 (−42.8 to 16.8)	
Sleeve	0.44 (0.36 to 0.54)	−19.4 (−30.9 to −7.9)	
Bypass	0.87 (0.47 to 1.60)	−11.4 (−61.1 to 38.3)	
*Personality disorders, n = 2085*			0.33
Restrictive	1.04 (0.72 to 1.49)	1.3 (−12.5 to 15.1)	
Sleeve	1.33 (1.21 to 1.47)	10.2 (6.4 to 14.1)	
Bypass	1.18 (0.89 to 1.55)	9.5 (−7.6 to 26.5)	
*Any psychiatric diagnosis, n = 15,211*			<0.0001
Restrictive	0.78 (0.68 to 0.89)	−11.8 (−18.2 to −5.5)	
Sleeve	1.16 (1.12 to 1.20)	8.1 (6.0 to 10.1)	
Bypass	1.66 (1.50 to 1.84)	42.6 (31.6 to 53.5)	
*Admission as a psychiatric inpatient, n = 6855*			<0.0001
Restrictive	0.77 (0.64 to 0.93)	−8.6 (−15.1 to −2.2)	
Sleeve	1.23 (1.17 to 1.30)	8.6 (6.3 to 10.8)	
Bypass	1.50 (1.28 to 1.76)	21.4 (11.7 to 31.1)	

*Note*: *n* in column 1 refers to the number of patients included in each fixed‐effects analysis.

Rate differences were calculated to give a sense of how many additional/fewer hospital admissions were estimated for each outcome post surgery (Table 3). To illustrate, restrictive procedures and sleeve gastrectomy were associated with reductions of 40.9 and 11.3 admissions for self‐harm (respectively) per 1000 person years. Admissions with substance‐use disorder increased by 17.5 (restrictive), 41.5 (sleeve), and 90.1 (bypass) admissions per 1000 person years.

Patterns were broadly similar for secondary outcomes, when analyses were limited to patients whose surgery took place after July 1, 2013 (Table 4 and Supporting Information Table S3). Rates of admission with self‐harm, mood disorders, and as a psychiatric inpatient no longer differed by surgery type, and all procedure types were associated with an increase in admission with anxiety disorders. Similar results were observed when the analysis was restricted to patients who did not have revisional bariatric surgery following their index procedure (Table 5 and Supporting Information Table S4).

**TABLE 4:** Presurgery and postsurgery hospitalization for secondary outcomes, fixed‐effects rate ratios, and rate differences (analysis of data from July 2013 onward)

Outcome by type of bariatric surgery	Fixed‐effects rate ratio (95% CI)	Fixed‐effects rate difference per 1000 person years (95% CI)	*p* value for interaction
*Self‐harm, n = 2445*			0.30
Restrictive	0.76 (0.44 to 1.31)	−10.1 (−34.9 to 14.6)	
Sleeve	0.98 (0.89 to 1.09)	−1.5 (−11.3 to 8.2)	
Bypass	1.17 (0.89 to 1.52)	12.6 (−10.6 to 35.8)	
*Substance‐use disorder, n = 4098*			0.009
Restrictive	2.11 (1.49 to 2.99)	26.1 (5.2 to 46.9)	
Sleeve	2.49 (2.32 to 2.66)	51.4 (43.5 to 59.2)	
Bypass	3.25 (2.74 to 3.84)	91.7 (58.9 to 124.6)	
*Schizophrenia disorders, n = 480*			0.61
Restrictive	1.25 (0.71 to 2.17)	92.9 (−203.4 to 389.1)	
Sleeve	1.35 (1.11 to 1.63)	21.0 (6.0 to 36.0)	
Bypass	1.00 (0.57 to 1.75)	0.1 (−37.3 to 37.5)	
*Mood disorders, n = 5222*			0.07
Restrictive	0.91 (0.68 to 1.23)	−2.4 (−10.3 to 5.5)	
Sleeve	1.20 (1.13 to 1.28)	8.3 (5.3 to 11.3)	
Bypass	1.36 (1.15 to 1.60)	15.7 (6.3 to 25.1)	
*Anxiety disorders, n = 6106*			<0.0001
Restrictive	1.37 (1.04 to 1.80)	12.4 (−0.2 to 25.1)	
Sleeve	1.22 (1.15 to 1.30)	10.5 (7.2 to 13.8)	
Bypass	1.75 (1.51 to 2.04)	34.5 (21.7 to 47.2)	
*Behavioral disorders, n = 463*			0.07
Restrictive	0.26 (0.08 to 0.80)	−0.0 (−0.1 to 0.1)	
Sleeve	0.51 (0.38 to 0.68)	−10.3 (−19.7 to −0.9)	
Bypass	1.04 (0.53 to 2.04)	4.1 (−68.4 to 76.5)	
*Personality disorders, n = 1379*			0.52
Restrictive	1.28 (0.70 to 2.34)	7.7 (−13.3 to 28.7)	
Sleeve	1.53 (1.36 to 1.72)	17.5 (11.7 to 23.2)	
Bypass	1.29 (0.94 to 1.75)	12.7 (−4.4 to 29.8)	
*Any psychiatric diagnosis, n = 10,437*			<0.0001
Restrictive	1.17 (0.95 to 1.43)	6.0 (−2.4 to 14.4)	
Sleeve	1.32 (1.27 to 1.38)	16.3 (13.5 to 19.1)	
Bypass	1.77 (1.59 to 1.98)	45.9 (34.0 to 57.8)	
*Admission as a psychiatric inpatient, n = 4589*			0.15
Restrictive	1.12 (0.84 to 1.51)	4.1 (−6.6 to 14.7)	
Sleeve	1.44 (1.35 to 1.54)	16.5 (13.3 to 19.8)	
Bypass	1.59 (1.33 to 1.89)	23.9 (13.2 to 34.7)	

*Note*: Sensitivity analysis on data from July 2013 onward. *n* in column 1 refers to the number of patients included in each fixed‐effects analysis.

**TABLE 5:** Presurgery and postsurgery hospitalization for secondary outcomes, fixed‐effects rate ratios, and rate differences (participants without surgery revision)

Outcome by type of bariatric surgery	Fixed‐effects rate ratio (95% CI)	Fixed‐effects rate difference per 1000 person years (95% CI)	*p* value for interaction
*Self‐harm, n = 2965*			0.07
Restrictive	0.65 (0.42 to 1.02)	−26.1 (−64.8 to 12.6)	
Sleeve	0.90 (0.82 to 0.98)	−9.4 (−17.1 to −1.6)	
Bypass	1.14 (0.89 to 1.45)	11.1 (−10.8 to 33.0)	
*Substance‐use disorder, n = 5073*			<0.0001
Restrictive	1.63 (1.16 to 2.29)	21.9 (1.5 to 42.2)	
Sleeve	2.25 (2.11 to 2.39)	41.6 (35.6 to 47.6)	
Bypass	3.14 (2.67 to 3.69)	89.4 (59.3 to 119.5)	
*Schizophrenia disorders, n = 661*			0.31
Restrictive	0.59 (0.31 to 1.13)	−42.9 (−110.1 to 24.2)	
Sleeve	1.00 (0.84 to 1.17)	−0.3 (−11.2 to 10.6)	
Bypass	1.00 (0.61 to 1.65)	0.2 (−37.9 to 38.2)	
*Mood disorders, n = 6582*			<0.0001
Restrictive	0.60 (0.46 to 0.77)	−13.9 (−22.4 to −5.4)	
Sleeve	1.02 (0.96 to 1.07)	0.7 (−1.8 to 3.2)	
Bypass	1.17 (1.00 to 1.36)	8.7 (−0.1 to 17.5)	
*Anxiety disorders, n = 7388*			<0.0001
Restrictive	0.84 (0.65 to 1.09)	−4.4 (−11.2 to 2.5)	
Sleeve	1.15 (1.09 to 1.21)	6.4 (3.8 to 9.0)	
Bypass	1.61 (1.40 to 1.87)	30.6 (18.6 to 42.6)	
*Behavioral disorders, n = 591*			0.05
Restrictive	0.45 (0.19 to 1.08)	−0.0 (−0.1 to 0.1)	
Sleeve	0.37 (0.29 to 0.47)	−24.3 (−41.6 to −7.0)	
Bypass	0.87 (0.46 to 1.63)	−12.0 (−66.8 to 42.7)	
*Personality disorders, n = 1701*			0.38
Restrictive	1.12 (0.63 to 2.00)	2.5 (−10.5 to 15.4)	
Sleeve	1.35 (1.21 to 1.50)	11.1 (6.7 to 15.4)	
Bypass	1.10 (0.83 to 1.47)	6.1 (−12.2 to 24.4)	
*Any psychiatric diagnosis, n = 12,834*			<0.0001
Restrictive	0.79 (0.65 to 0.95)	−8.2 (−15.0 to −1.4)	
Sleeve	1.16 (1.12 to 1.21)	8.4 (6.1 to 10.6)	
Bypass	1.59 (1.43 to 1.77)	38.0 (27.2 to 48.8)	
*Admission as a psychiatric inpatient, n = 5702*			0.003
Restrictive	0.80 (0.60 to 1.07)	−5.4 (−12.6 to 1.9)	
Sleeve	1.24 (1.17 to 1.31)	8.9 (6.4 to 11.5)	
Bypass	1.43 (1.21 to 1.68)	18.9 (9.2 to 28.7)	

*Note*: Sensitivity analysis on participants without bariatric surgery revision. *n* in column 1 refers to the number of patients included in each fixed‐effects analysis.

## DISCUSSION

In this cohort of over 120,000 bariatric surgery patients followed for up to 19 years, we examined whether the risk of adverse mental health outcomes changed after sleeve gastrectomy and whether these risks differed by type of bariatric procedure. Our principal findings were that rates of suicide did not differ among procedures, whereas changes from presurgery to postsurgery in rates of hospital admission with several psychiatric diagnoses differed by surgery type. Rates of admission with self‐harm declined after restrictive and sleeve procedures; admission with anxiety disorders, any psychiatric diagnosis, and as a psychiatric inpatient increased after sleeve gastrectomy and gastric bypass (but not restrictive procedures); and admissions with substance‐use disorder were higher after all procedure types compared with before surgery, with the highest risk associated with gastric bypass.

Our findings are consistent with prior research that has identified increased risks of suicide, mental health‐service use, and substance‐use disorder after gastric bypass [4, 5, 6] and extend this literature by examining mental health outcomes after sleeve gastrectomy and comparing within‐person changes in rates of these outcomes before and after different bariatric procedures. The results of the present study are in line with earlier, smaller studies that have found higher risks of adverse mental health outcomes in patients who had undergone bariatric surgery, including sleeve gastrectomy, compared with nonsurgical cohorts with obesity [12, 13].

A longitudinal study examining a separate cohort of Australian patients [6] reported that mental health‐service use (including outpatient clinic, emergency department attendance, and hospital admission) was increased after bariatric surgery, and that sleeve gastrectomy and gastric bypass (but not procedures coded as “gastric reduction”) were associated with increased risks of emergency department attendance with deliberate self‐harm or suicidal ideation [6]. Like that study [6], we used ACHI codes for bariatric surgery to define the patient cohort. Although both studies found increased rates of hospitalization for mental health outcomes after surgery, there were some major methodological differences that might explain the contrasting findings regarding self‐harm admissions. First, our analysis focused on changes in outcomes within individuals (rather than mean changes within the cohort) undergoing each procedure, and we separated the code for “gastric reduction” into restrictive procedures or sleeve gastrectomy based on Australian trends in bariatric surgery during the study period. The concordance between results of our main analysis and the analysis limited to surgeries performed after the change in ACHI coding supports the validity of this approach.

Our finding of an increase in hospital admissions with substance‐use disorder after all surgery types, with a higher risk for gastric bypass compared with sleeve gastrectomy and restrictive procedures, is consistent with some previous studies, whereas others have found comparably high risks after sleeve gastrectomy and gastric bypass [14, 15, 16]. Owing to the different outcomes and analysis methods, direct comparisons among the previous and current studies are not possible. Nonetheless, their notable similarity is the finding of a higher risk of adverse mental health outcomes after sleeve gastrectomy and gastric bypass compared with preoperatively or after restrictive bariatric procedures. A recent matched cohort study also reported a higher risk of substance‐use disorder and self‐harm in patients with class 1 obesity who underwent sleeve gastrectomy compared with an intensive lifestyle intervention [13]. Although overall quality of life and symptoms of depression usually improve after bariatric surgery, together these results provide a consistent indication of an increased risk of certain adverse psychiatric outcomes associated with sleeve gastrectomy, as well as gastric bypass.

Changes in mental health after bariatric surgery are not surprising. Commonly reported overall improvements in symptoms of depression and psychological wellbeing [2] may reflect improvements in complications of obesity, health‐related quality of life, and the experience of obesity‐related stigma after weight loss. Conversely, the high prevalence of depression, anxiety, and history of trauma in bariatric surgery candidates compared with the general population, unmet expectations of a transformative effect of weight loss on quality of life, changes in interpersonal relationships, the impact of body dissatisfaction and excess skin after substantial weight loss, and complications of surgery could contribute to adverse psychological health after surgery [6, 7, 17]. However, these factors do not explain differences in psychiatric outcomes among procedures.

The patient cohorts undergoing each procedure may be distinct in their vulnerability to the impact of weight loss on psychological wellbeing because the choice of operation is primarily determined by patient characteristics as they relate to risks and benefits, as well as personal preferences of patients and surgeons. Numerous differences have been noted among gastric bypass patients and those undergoing sleeve gastrectomy or gastric banding, with respect to preoperative sociodemographic, psychological and behavioral characteristics, medical comorbidities, postoperative eating behaviors, relationship with food, and satisfaction with surgical outcomes, despite similarities in age, sex, and body mass index (BMI) 1[18, 19].

Additionally, the observation that partial gastrectomy in non‐bariatric populations (e.g., for peptic ulcer disease) is also associated with psychiatric adverse outcomes, including suicide and alcohol‐use disorder [20], raises the possibility that anatomical or functional changes resulting from gastrointestinal surgery may contribute to alterations in mental health. Preclinical data suggest links among gut‐derived hormones, microbial and bile acid profiles (which are variably affected by different surgery types), and mood and behaviors [8], which need to be confirmed in mechanistic studies. Changes in gastrointestinal transit time and surface area available for absorption of medications, other drugs, and alcohol are also likely to differ among procedures. Reduction in therapeutic effect or exposure to antidepressant and/or antipsychotic medications, a higher, faster peak in breath and/or blood alcohol concentration, and greater rewarding properties of alcohol have been reported after gastric bypass [21, 22], but comparable information is lacking or inconsistent for sleeve gastrectomy and restrictive procedures [23, 24, 25].

Examination of the contribution of these factors to mental health outcomes after different bariatric surgeries should be prioritized in future research. Given the rarity of adverse psychiatric outcomes, a randomized trial of the scale required to detect differences among procedures is unlikely ever to be conducted, but collaborative prospective cohort studies with standardized collection of detailed demographic, psychosocial, and behavioral data before and after surgery may more feasibly allow determination of risk factors and early markers of deterioration.

A strength of this study is inclusion of data from more than half of bariatric surgeries performed in adults in Australia over a 20‐year period. To our knowledge, this represents the largest analysis to date of mental health outcomes after sleeve gastrectomy, the most common bariatric procedure worldwide. Furthermore, our fixed‐effects analysis of within‐person changes in outcomes for patients who underwent each procedure has the advantage (compared with mean cohort changes) of accounting for unobserved (i.e., unavailable in the data set) confounding factors that remained constant within individuals.

This study also has several limitations. In particular, the available data did not allow for detailed characterization of the groups at baseline or during follow‐up. Although we controlled for several covariates in our primary analysis, there remains a risk of residual confounding from factors not captured in our population‐based data sets, such as medication prescription and adherence, alcohol and substance use, and pre‐ and postoperative psychological assessment and support. Weight and height were not available, making it impossible to account for weight or BMI at baseline or during follow‐up as confounding variables in mental health outcomes, or to create a BMI‐matched nonsurgical control cohort with which to compare outcomes during the study period. The relationship between postsurgical weight trajectory and mental health outcomes is not straightforward. Although it has been proposed that insufficient weight loss or weight regain, with less‐than‐expected improvement in health or recurrence of obesity‐related complications, may adversely affect mental health by contributing to a sense of disappointment or failure [26], others have found no relationship between weight loss and depression [27] and similar or greater weight loss in people who died by suicide or had a self‐harm event compared with those who did not [4]. The low overall incidence of suicide limited our ability to compare this outcome among surgery types. Psychiatric outcomes that were managed in primary care, community mental health services, or in emergency departments that did not result in hospitalization were not captured in the available data. Therefore, their rates are likely to be underestimated because only one‐third of psychiatric emergency department presentations result in hospital admission in Australia [28]. The lack of unique ACHI codes for gastroplasty, gastric banding, and sleeve gastrectomy prior to 2013 is another limitation; however, the reliability of our main findings is supported by the similar results for surgeries performed only after this date. Finally, it is both a strength and a limitation that the cohort included patients who underwent surgery in both publicly funded and private health care settings. This increases the variability of pre‐ and postoperative psychological care (which was not able to be determined from the available data) but also increases the generalizability of the findings within regions with similar health care systems.

Current clinical practice guidelines recommend formal psychosocial evaluation by a qualified practitioner for all patients [29]; however, although there is a high prevalence of mental illness in people with obesity seeking bariatric surgery, only a small minority (including those without preexisting conditions) will experience a decline in mental health after surgery, whereas, overall, most will experience significant benefits. As such, research to understand the mechanisms underlying these adverse effects is needed in order to develop evidence‐based guidance on how to identify and follow‐up at‐risk individuals, detect deterioration early in its course, and intervene effectively to prevent or reduce these risks.

## CONCLUSION

Different associations between bariatric surgeries and hospitalization with psychiatric diagnoses by procedure type might indicate distinct vulnerabilities among cohorts of patients who undergo each procedure or that anatomical and/or functional changes induced by different procedures may contribute to effects on mental health. Understanding these mechanisms will enable development of evidence‐based management strategies.

## FUNDING INFORMATION

This work received no specific funding. Priya Sumithran is supported by an Investigator Grant from the National Health and Medical Research Council (1178482). Robyn M. Brown is supported by an Australian Research Council Discovery Early Career Researcher Award (DE190101244). Matthew J. Spittal is a recipient of an Australian Research Council Future Fellowship (FT180100075) funded by the Australian Government.

## CONFLICT OF INTEREST STATEMENT

Priya Sumithran has received grants from the National Health and Medical Research Council (paid to institution) and coauthored manuscripts assisted by medical writing from Novo Nordisk A/S. Ian D. Caterson reports personal fees from Novo Nordisk A/S, including for lectures and participation on a trial steering committee, and grants or contracts for clinical trial involvement from Eli Lilly and Company, Boehringer Ingelheim, and the New South Wales Office of Health and Medical Research outside the submitted work. Wendy A. Brown reports personal fees from W.L. Gore & Associates, Inc., Merck Sharp & Dohme, Novo Nordisk A/S, and Pfizer for participation in lectures and advisory boards; and grants from Johnson & Johnson, W.L. Gore & Associates, Novo Nordisk A/S, Medtronic, Applied Medical, Myerton, and National Health and Medical Research Council and Commonwealth Government of Australia. The other authors declared no conflict of interest.

## Supporting information


**Table S1.** Australian Classification of Health Interventions (ACHI) bariatric surgery codes.
**Table S2.** Crude rates from entire cohort for hospitalization for secondary outcomes pre‐ and post‐surgery.
**Table S3.** Crude rates from entire cohort for hospitalization for secondary outcomes pre‐ and post‐surgery.

## Data Availability

Individual participant data that underlie the results reported in this paper will not be available for sharing.
