# Supplementary material for: Incidence of adverse mental health outcomes after sleeve gastrectomy compared with gastric bypass and restrictive bariatric procedures: a retrospective cohort study
Source: Obesity (Silver Spring). 2023 Jun 27;31(7):1913–23. doi: 10.1002/oby.23757 (PMC10946809; doi:10.1002/oby.23757)
Supplement: Supplementary file 1 — Table S1. Australian Classification of Health Interventions (ACHI) bariatric surgery codes. Table S2. Crude rates from entire cohort for hospitalization for secondary outcomes pre‐ and post‐surgery. Table S3. Crude rates from entire cohort for hospitalization for secondary outcomes pre‐ and post‐surgery. [file OBY-31-1913-s001.docx]

**Incidence of adverse mental health outcomes after sleeve gastrectomy compared with gastric bypass and restrictive bariatric procedures: a retrospective cohort study**

Priya Sumithran PhD (ORCID 0000-0002-9576-1050), Leo Roberts PhD, Ian D Caterson PhD, Robyn M Brown PhD, Matthew J Spittal* PhD, Wendy A Brown* PhD

**Correspondence to:**

Dr Priya Sumithran

Level 4, Clinical Sciences Building, St Vincent’s Hospital

Fitzroy, Victoria 3065 Australia

T: +61 3 9231 2592

E: priyas@unimelb.edu.au

**Table S1: Australian Classification of Health Interventions (ACHI) bariatric surgery codes**

| **ACHI Code** | **Bariatric Surgery Description** | **Classification for this analysis** |
| --- | --- | --- |
| Prior to July 2013 (Prior to 8th Edition) | |  |
| **30511-00** | Gastric reduction | Pre-2007 Restrictive  2007-2013 Sleeve gastrectomy |
| **30511-01** | Laparoscopic gastric reduction | Pre-2007 Restrictive  2007-2013 Sleeve gastrectomy |
| **30512-00** | Gastric bypass | Gastric bypass |
| **30512-01** | Laparoscopic biliopancreatic diversion | Other malabsorptive |
| **30512-02** | Biliopancreatic diversion | Other malabsorptive |
| **30518-00 with (E66)** | Partial gastrectomy (with a co-diagnosis of obesity) | Pre-2007 omitted  2007-2013 Sleeve gastrectomy |
| **90950–00** | Insertion of gastric bubble or balloon | Temporary endoscopic |
| From July 2013 (8th Edition) | |  |
| **30511-09** | Laparoscopic sleeve gastrectomy | Sleeve gastrectomy |
| **30511-10** | Sleeve gastrectomy | Sleeve gastrectomy |
| **30511-02** | Laparoscopic adjustable gastric banding | Restrictive |
| **30511-03** | Laparoscopic nonadjustable gastric banding | Restrictive |
| **30511-04** | Adjustable gastric banding | Restrictive |
| **30511-05** | Nonadjustable gastric banding | Restrictive |
| **30512-03** | Laparoscopic gastric bypass | Gastric bypass |
| **30512-00** | Gastric bypass | Gastric bypass |
| **30512-01** | Laparoscopic biliopancreatic diversion | Other malabsorptive |
| **30512-02** | Biliopancreatic diversion | Other malabsorptive |
| **30511-06** | Laparoscopic gastroplasty | Restrictive |
| **30511-07** | Endoscopic gastroplasty | Restrictive |
| **30511-08** | Gastroplasty | Restrictive |
| **90940-00** | Duodenal-jejunal bypass | Other malabsorptive |
| **90941-00** | Ileal interposition | Other malabsorptive |
| **90950-00** | Insertion of gastric balloon | Temporary endoscopic |

**Table S2: Crude rates from entire cohort for hospitalization for secondary outcomes pre- and post-surgery**

|  | **Type of bariatric surgery** | **Number of events pre-surgery** | **Crude rate pre-surgery (95% CI) per 1,000 person years** | **Number of events post-surgery** | **Crude rate post-surgery (95% CI) per 1,000 person years** |
| --- | --- | --- | --- | --- | --- |
| Self-harm |  |  |  |  |  |
|  | Restrictive | 131 | 5.17 (4.32 to 6.13) | 278 | 4.93 (4.37 to 5.54) |
|  | Sleeve | 3,748 | 5.54 (5.37 to 5.72) | 2,203 | 5.32 (5.10 to 5.55) |
|  | Bypass | 520 | 6.55 (6.00 to 7.13) | 258 | 9.24 (8.15 to 10.44) |
| Substance use disorder |  |  |  |  |  |
|  | Restrictive | 344 | 13.56 (12.17 to 15.08) | 1,454 | 25.78 (24.47 to 27.14) |
|  | Sleeve | 10,433 | 15.43 (15.13 to 15.72) | 14,383 | 34.73 (34.17 to 35.30) |
|  | Bypass | 1,207 | 15.19 (14.35 to 16.08) | 1,786 | 63.96 (61.02 to 66.99) |
| Schizophrenia disorders |  |  |  |  |  |
|  | Restrictive | 218 | 8.60 (7.49 to 9.82) | 436 | 7.73 (7.02 to 8.49) |
|  | Sleeve | 3,570 | 5.28 (5.11 to 5.45) | 4,270 | 10.31 (10.00 to 10.63) |
|  | Bypass | 547 | 6.89 (6.32 to 7.49) | 247 | 8.85 (7.78 to 10.02) |
| Mood disorders |  |  |  |  |  |
|  | Restrictive | 1,690 | 66.64 (63.50 to 69.89) | 3,872 | 68.65 (66.51 to 70.85) |
|  | Sleeve | 32,008 | 47.33 (46.81 to 47.85) | 33,549 | 81.01 (80.15 to 81.88) |
|  | Bypass | 4,308 | 54.23 (52.62 to 55.87) | 3,323 | 119.00 (114.99 to 123.11) |
| Anxiety disorders |  |  |  |  |  |
|  | Restrictive | 840 | 33.12 (30.92 to 35.44) | 2,926 | 51.88 (50.02 to 53.79) |
|  | Sleeve | 22,567 | 33.37 (32.93 to 33.81) | 23,426 | 56.57 (55.85 to 57.30) |
|  | Bypass | 3,147 | 39.61 (38.24 to 41.02) | 2,842 | 101.77 (98.06 to 105.58) |
| Behavioural disorders |  |  |  |  |  |
|  | Restrictive | 37 | 1.46 (1.03 to 2.01) | 63 | 1.12 (0.86 to 1.43) |
|  | Sleeve | 753 | 1.11 (1.04 to 1.20) | 317 | 0.77 (0.68 to 0.85) |
|  | Bypass | 110 | 1.38 (1.14 to 1.67) | 46 | 1.65 (1.21 to 2.20) |
| Personality disorders |  |  |  |  |  |
|  | Restrictive | 235 | 9.27 (8.12 to 10.53) | 963 | 17.07 (16.01 to 18.19) |
|  | Sleeve | 9,954 | 14.72 (14.43 to 15.01) | 9,593 | 23.16 (22.70 to 23.63) |
|  | Bypass | 1,190 | 14.98 (14.14 to 15.86) | 906 | 32.44 (30.37 to 34.63) |
| Any psychiatric diagnosis |  |  |  |  |  |
|  | Restrictive | 2,579 | 101.69 (97.80 to 105.69) | 6,298 | 111.67 (108.93 to 114.46) |
|  | Sleeve | 55,246 | 81.69 (81.01 to 82.37) | 56,402 | 136.20 (135.08 to 137.33) |
|  | Bypass | 7,415 | 93.34 (91.23 to 95.49) | 5,899 | 211.24 (205.89 to 216.71) |
| Admission as a psychiatric inpatient |  |  |  |  |  |
|  | Restrictive | 1,996 | 78.70 (75.29 to 82.23) | 4,858 | 86.13 (83.73 to 88.59) |
|  | Sleeve | 41,121 | 60.80 (60.21 to 61.39) | 43,943 | 106.11 (105.12 to 107.11) |
|  | Bypass | 5,427 | 68.32 (66.51 to 70.16) | 4,196 | 150.26 (145.75 to 154.88) |

Notes: Crude rates calculated using the person time from the whole sample.

**Table S3: Crude rates from entire cohort for hospitalization for secondary outcomes pre- and post-surgery. Data from July 2013 onwards**

|  | **Type of bariatric surgery** | **Number of events pre-surgery** | **Crude rate pre-surgery (95% CI) per 1,000 person years** | **Number of events post-surgery** | **Crude rate post-surgery (95% CI) per 1,000 person years** |
| --- | --- | --- | --- | --- | --- |
| Self-harm |  |  |  |  |  |
|  | Restrictive | 59 | 3.38 (2.57 to 4.36) | 46 | 4.04 (2.96 to 5.39) |
|  | Sleeve | 2,992 | 5.21 (5.03 to 5.40) | 1,125 | 5.54 (5.22 to 5.88) |
|  | Bypass | 478 | 6.28 (5.73 to 6.87) | 138 | 7.24 (6.09 to 8.56) |
| Substance use disorder |  |  |  |  |  |
|  | Restrictive | 188 | 10.76 (9.28 to 12.41) | 478 | 41.99 (38.31 to 45.93) |
|  | Sleeve | 8,320 | 14.49 (14.18 to 14.81) | 6,416 | 31.61 (30.84 to 32.39) |
|  | Bypass | 1,120 | 14.72 (13.87 to 15.61) | 1,015 | 53.28 (50.05 to 56.66) |
| Schizophrenia disorders |  |  |  |  |  |
|  | Restrictive | 97 | 5.55 (4.50 to 6.77) | 124 | 10.89 (9.06 to 12.99) |
|  | Sleeve | 2,535 | 4.42 (4.25 to 4.59) | 1,618 | 7.97 (7.59 to 8.37) |
|  | Bypass | 517 | 6.80 (6.22 to 7.41) | 151 | 7.93 (6.71 to 9.30) |
| Mood disorders |  |  |  |  |  |
|  | Restrictive | 844 | 48.31 (45.10 to 51.68) | 822 | 72.21 (67.35 to 77.32) |
|  | Sleeve | 24,788 | 43.18 (42.64 to 43.72) | 14,729 | 72.56 (71.39 to 73.74) |
|  | Bypass | 4,029 | 52.96 (51.33 to 54.62) | 2,273 | 119.31 (114.46 to 124.32) |
| Anxiety disorders |  |  |  |  |  |
|  | Restrictive | 362 | 20.72 (18.64 to 22.97) | 805 | 70.71 (65.91 to 75.77) |
|  | Sleeve | 18,133 | 31.59 (31.13 to 32.05) | 10,486 | 51.66 (50.67 to 52.65) |
|  | Bypass | 3,040 | 39.96 (38.55 to 41.40) | 2,100 | 110.23 (105.57 to 115.05) |
| Behavioural disorders |  |  |  |  |  |
|  | Restrictive | 25 | 1.43 (0.93 to 2.11) | <5 | 0.35 (0.10 to 0.90) |
|  | Sleeve | 544 | 0.95 (0.87 to 1.03) | 163 | 0.80 (0.68 to 0.94) |
|  | Bypass | 102 | 1.34 (1.09 to 1.63) | 33 | 1.73 (1.19 to 2.43) |
| Personality disorders |  |  |  |  |  |
|  | Restrictive | 68 | 3.89 (3.02 to 4.93) | 134 | 11.77 (9.86 to 13.94) |
|  | Sleeve | 8,347 | 14.54 (14.23 to 14.86) | 4,613 | 22.72 (22.07 to 23.39) |
|  | Bypass | 1,127 | 14.81 (13.96 to 15.70) | 669 | 35.12 (32.51 to 37.88) |
| Any psychiatric diagnosis |  |  |  |  |  |
|  | Restrictive | 1,248 | 71.43 (67.52 to 75.51) | 1,526 | 134.05 (127.41 to 140.95) |
|  | Sleeve | 42,970 | 74.85 (74.15 to 75.56) | 24,615 | 121.26 (119.75 to 122.78) |
|  | Bypass | 6,993 | 91.92 (89.77 to 94.10) | 3,869 | 203.09 (196.74 to 209.59) |
| Admission as a psychiatric inpatient |  |  |  |  |  |
|  | Restrictive | 1,025 | 58.67 (55.13 to 62.37) | 1,272 | 111.74 (105.68 to 118.05) |
|  | Sleeve | 31,926 | 55.61 (55.01 to 56.23) | 19,025 | 93.72 (92.39 to 95.06) |
|  | Bypass | 5,188 | 68.19 (66.35 to 70.07) | 2,918 | 153.17 (147.66 to 158.83) |

Notes: Crude rates calculated using the person time from the whole sample.

**Table S4: Crude rates from entire cohort for hospitalization for secondary outcomes pre- and post-surgery. Data from participants without bariatric surgery revision**

|  | **Type of bariatric surgery** | **Number of events pre-surgery** | **Crude rate pre-surgery (95% CI) per 1,000 person years** | **Number of events post-surgery** | **Crude rate post-surgery (95% CI) per 1,000 person years** |
| --- | --- | --- | --- | --- | --- |
| Self-harm |  |  |  |  |  |
|  | Restrictive | 59 | 3.38 (2.57 to 4.36) | 46 | 4.04 (2.96 to 5.39) |
|  | Sleeve | 2,992 | 5.21 (5.03 to 5.40) | 1,125 | 5.54 (5.22 to 5.88) |
|  | Bypass | 478 | 6.28 (5.73 to 6.87) | 138 | 7.24 (6.09 to 8.56) |
| Substance use disorder |  |  |  |  |  |
|  | Restrictive | 188 | 10.76 (9.28 to 12.41) | 478 | 41.99 (38.31 to 45.93) |
|  | Sleeve | 8,320 | 14.49 (14.18 to 14.81) | 6,416 | 31.61 (30.84 to 32.39) |
|  | Bypass | 1,120 | 14.72 (13.87 to 15.61) | 1,015 | 53.28 (50.05 to 56.66) |
| Schizophrenia disorders |  |  |  |  |  |
|  | Restrictive | 97 | 5.55 (4.50 to 6.77) | 124 | 10.89 (9.06 to 12.99) |
|  | Sleeve | 2,535 | 4.42 (4.25 to 4.59) | 1,618 | 7.97 (7.59 to 8.37) |
|  | Bypass | 517 | 6.80 (6.22 to 7.41) | 151 | 7.93 (6.71 to 9.30) |
| Mood disorders |  |  |  |  |  |
|  | Restrictive | 844 | 48.31 (45.10 to 51.68) | 822 | 72.21 (67.35 to 77.32) |
|  | Sleeve | 24,788 | 43.18 (42.64 to 43.72) | 14,729 | 72.56 (71.39 to 73.74) |
|  | Bypass | 4,029 | 52.96 (51.33 to 54.62) | 2,273 | 119.31 (114.46 to 124.32) |
| Anxiety disorders |  |  |  |  |  |
|  | Restrictive | 362 | 20.72 (18.64 to 22.97) | 805 | 70.71 (65.91 to 75.77) |
|  | Sleeve | 18,133 | 31.59 (31.13 to 32.05) | 10,486 | 51.66 (50.67 to 52.65) |
|  | Bypass | 3,040 | 39.96 (38.55 to 41.40) | 2,100 | 110.23 (105.57 to 115.05) |
| Behavioural disorders |  |  |  |  |  |
|  | Restrictive | 25 | 1.43 (0.93 to 2.11) | <5 | 0.35 (0.10 to 0.90) |
|  | Sleeve | 544 | 0.95 (0.87 to 1.03) | 163 | 0.80 (0.68 to 0.94) |
|  | Bypass | 102 | 1.34 (1.09 to 1.63) | 33 | 1.73 (1.19 to 2.43) |
| Personality disorders |  |  |  |  |  |
|  | Restrictive | 68 | 3.89 (3.02 to 4.93) | 134 | 11.77 (9.86 to 13.94) |
|  | Sleeve | 8,347 | 14.54 (14.23 to 14.86) | 4,613 | 22.72 (22.07 to 23.39) |
|  | Bypass | 1,127 | 14.81 (13.96 to 15.70) | 669 | 35.12 (32.51 to 37.88) |
| Any psychiatric diagnosis |  |  |  |  |  |
|  | Restrictive | 1,248 | 71.43 (67.52 to 75.51) | 1,526 | 134.05 (127.41 to 140.95) |
|  | Sleeve | 42,970 | 74.85 (74.15 to 75.56) | 24,615 | 121.26 (119.75 to 122.78) |
|  | Bypass | 6,993 | 91.92 (89.77 to 94.10) | 3,869 | 203.09 (196.74 to 209.59) |
| Admission as a psychiatric inpatient |  |  |  |  |  |
|  | Restrictive | 1,025 | 58.67 (55.13 to 62.37) | 1,272 | 111.74 (105.68 to 118.05) |
|  | Sleeve | 31,926 | 55.61 (55.01 to 56.23) | 19,025 | 93.72 (92.39 to 95.06) |
|  | Bypass | 5,188 | 68.19 (66.35 to 70.07) | 2,918 | 153.17 (147.66 to 158.83) |

Notes: Crude rates calculated using the person time from the whole sample.
